# Evidence That Voltage Rather Than Resistance is Quantized in Breakdown of the Quantum Hall Effect

**DOI:** 10.6028/jres.101.019

**Published:** 1996

**Authors:** M. E. Cage

**Affiliations:** National Institute of Standards and Technology, Gaithersburg, MD 20899-0001

**Keywords:** breakdown, quantized dissipation, quantized resistance states, quantized voltage states, quantum Hall effect, quasi-elastic inter-Landau level scattering, two-dimensional electron gas

## Abstract

Quantized longitudinal voltage drops are observed along a length of a GaAs/AlGaAs heterostructure quantum Hall effect device at applied currents large enough for the device to be in the breakdown regime. The range of currents is extensive enough to demonstrate that it is the longitudinal voltage that is quantized, rather than the longitudinal resistance. A black-box and a quasi-elastic inter-Landau level scattering (QUILLS) model are then employed to calculate the fraction of electrons making transitions into higher Landau levels, the transition rates, and the maximum electric field across the device.

## 1. Introduction

The integer quantum Hall effect [1–3] requires a fully quantized two-dimensional electron gas (2DEG). At low currents there is negligible dissipation within the interior of the 2DEG in the Hall plateau regions of high-quality devices. Within these regions the Hall resistance *R*_H_ of the *i* th plateau has the value *R*_H_(*i*) = *h*/(*e*^2^*i*), where *h* is the Planck constant, *e* is the elementary charge, and *i* is an integer. At high currents, however, energy dissipation can suddenly appear [4, 5]. This is often referred to as the breakdown regime of the quantum Hall effect.

Dissipative breakdown signals can be detected by measuring longitudinal voltage differences *V_x_* between potential probes placed along the side of a device, where *x* is the direction of current flow. Cage et al. [6–9] found examples where the curves of breakdown voltages *V_x_* plotted versus magnetic flux density *B* were definitely quantized. It could be, however, that it is the longitudinal resistance *R_x_* that is quantized, rather than *V_x_*, since *R_x_ = V_x_*/*I_x_*. Indeed, Bliek et al. [10] assumed a quantized *R_x_* in a phenomenological model to explain breakdown structures in their curves of *V_x_* versus *B* for samples with narrow constrictions. Also, although not quantized, Sachrajda et al. [11] assumed magnetic field and current dependent resistive channels along the sample to explain their breakdown data.

Knowing whether *R_x_* or *V_x_* is quantized does matter because it can help determine what mechanism best describes the breakdown phenomena. For example, the quasi-elastic inter-Landau level scattering (QUILLS) models of Heinonen, Taylor, and Girvin [12] and Eaves and Sheard [13] assume that *V_x_* is quantized.

It was not possible to determine which entity, *R_x_* or *V_x_*, was quantized in our previous experiments [6–9] because the critical currents for the advent of breakdown were high, and the range of source-drain currents *I*_SD_ = *I_x_* over which quantized breakdown occurred varied by only a few percent. It will be possible, however, to show in the present experiment that it is *V_x_* which is quantized because the range of currents is more extensive.

A black-box model [7–9] that is based on the conservation of energy will then be used to determine the fraction of electrons making transitions between Landau levels and the transition rates. In addition, the maximum electric field across the sample will be deduced from the QUILLS model of Eaves and Sheard [13].

## 2. Experiment

### 2.1 Device

The device is a GaAs/Al*_x_*Ga_1−_*_x_*As heterostructure^1^ grown by molecular beam epitaxy at AT&T Bell Laboratories, with *x* = 0.29 being the fraction of aluminum atoms replacing gallium atoms in the crystal. It is designated as GaAs(7), has a zero magnetic field mobility of about 100 000 cm^2^/(Vs) at 1.2 K, and exhibits excellent integral quantum Hall effect properties.

**Fig. 1:**
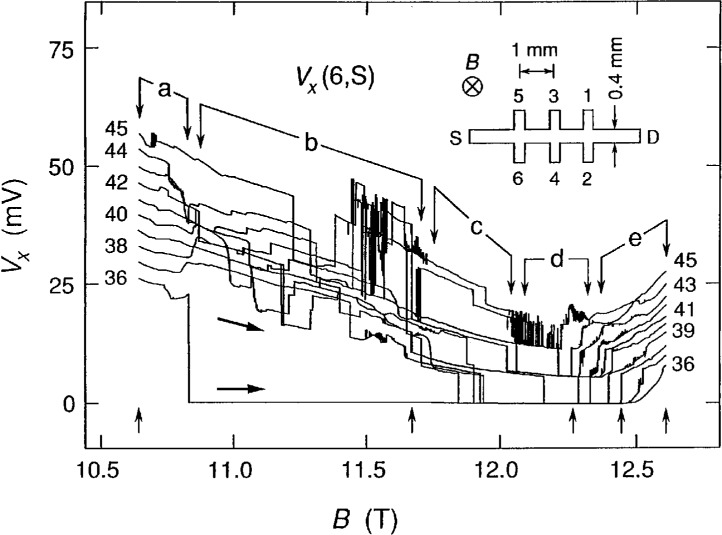
Ten sweeps of *V_x_*(6,S) versus *B* for the *i* = 2 plateau at 1.2 K with applied currents between +36 μA and +45 μA in 1 μA increments. The sweeps are in the direction of increasing *B*. The data have different characteristics in regions a through e. Upward arrows indicate magnetic flux density values for which calculations are made in Figs. 2 and 3. The inset shows the sample geometry and the magnetic field direction.

The inset of Fig. 1 shows the device geometry. It is 4.6 mm long and has a width *w* of 0.4 mm. The two outer Hall potential probe pairs are displaced from the central pair by ±1 mm. The magnetic flux density *B* is perpendicular to the device and points into the figure. Electrons enter at the upper left hand corner of the device and exit at the lower right hand corner for this magnetic field direction and current. Potential probes 2, 4, and 6 are near the potential of the source S, which is grounded. Probes 1, 3, and 5 are near the drain potential D, and have a positive potential relative to the source.

### 2.2 Longitudinal Voltage Versus Magnetic Flux Density

The dissipative voltages *V_x_* were measured between probes 6 and S, hereafter denoted as *V_x_*(6,S) ≡ *V*(6)−*V*(S). These two probes were chosen because breakdown occurred over a wide range of source-drain currents in this region of the device. The contact resistances were negligible, so they did not contribute to the *V_x_* signals. Normally we would also monitor the longitudinal voltage *V_x_*(5, S) on the opposite side of the device to assure that they were the same as *V_x_*(6, S), but in this case the *V_x_*(5, S) signal corresponds to integer quantum Hall voltages *V*_H_ = *R*_H_*I*_SD_, which were also observed on probe set *V*_H_(5, 6).

**Fig. 2:**
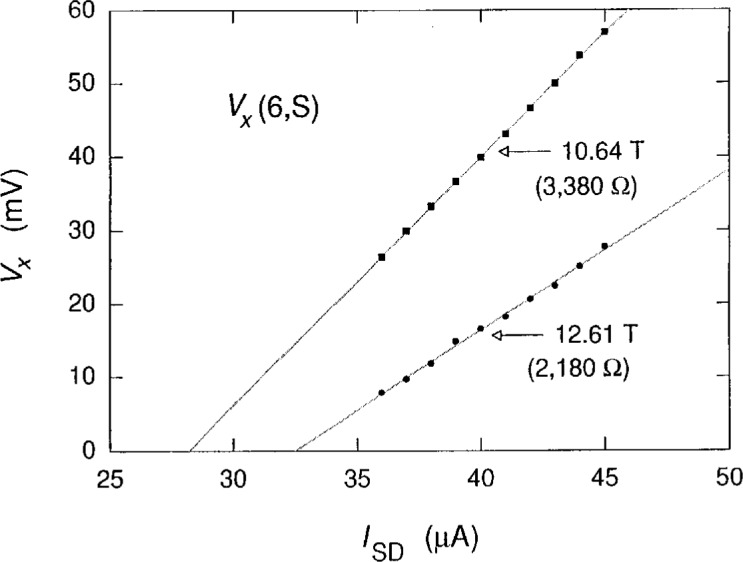
Plots of *V_x_*(6,S) versus *I*_SD_ at magnetic flux density values corresponding to the beginning and the end of the sweeps if Fig. 1. The resistance values in parentheses are the slopes of the straight lines fitted to the data. The sample has an ohmic behavior at these two magnetic fields.

Figure 1 shows ten sweeps of *V_x_*(6, S) versus the magnetic flux density *B* for the *i* =2 (12 906.4 Ω) quantized Hall resistance plateau at a temperature of 1.2 K for injected electron currents *I*_SD_ of +36 μA to +45 μA in 1 μA increments, where positive current corresponds to electrons entering the source and exiting the drain. Distinct changes in character of the *V_x_* signals occur in the five regions, a to e, in the figure. The signals in regions a and e have some structure, but mainly depend on the magnitude of the current. This current dependence was determined at magnetic flux density values of 10.64 T and 12.61 T (indicated in Fig. 1 by the upward arrows at the beginning and end of the sweeps) by plotting *V_x_* versus *I*_SD_ in Fig. 2. *V_x_* increases linearly with current, and Δ*V_x_/*Δ*I*_SD_ is 3.38 kΩ and 2.18 kΩ at 10.64 T and 12.61 T, respectively; so regions a and e basically exhibit an ohmic behavior. The data in region b clearly show discrete, well-defined voltage states, with some switching between states, but they have very little correlation with current. The data in region c are quantized, and have a current dependence. The signals in region d are quantized, and the quantization is current-independent to within 1 % over a current range that varies by 25 %. Therefore, dissipation in the middle region of the quantized Hall resistance, which happens to be the magnetic flux density regime that has the best developed breakdown quantization, involves a quantized voltage *V_x_* rather than a quantized resistance *R*_x_.

### 2.3 Critical Current

The critical current *I*_cr_ for which *V_x_*(6, S) is never zero across the magnetic field sweep is +40 μA. It was ±230 μA for *V_x_*(2, 4) on the same device [7, 8]. This could imply that the reduced critical current for *V_x_*(6, S) is due to an influence from the current emerging from the corner of the source, such as the heating-induced current instabilities proposed by Komiyama et al. [14]. Such a mechanism is not the reason for the reduced value of *I*_cr_ for *V_x_*(6, S), however, because *I*_cr_ was still only about −44 μA for the opposite current direction. We have not found a correlation of the critical current value with the location of *V_x_*. For instance, in a device designated as GaAs(2), *I*_cr_ was about ±20 μA at one end of the device for *V_x_*(6, S), ±87 μA for *V_x_*(4, 6), only ±21 μA for *V_x_*(2, 4), and the largest value ±134 μA for the other end, *V_x_*(D,1).

## 3. Analysis

### 3.1 Transition Rates

**Fig. 3:**
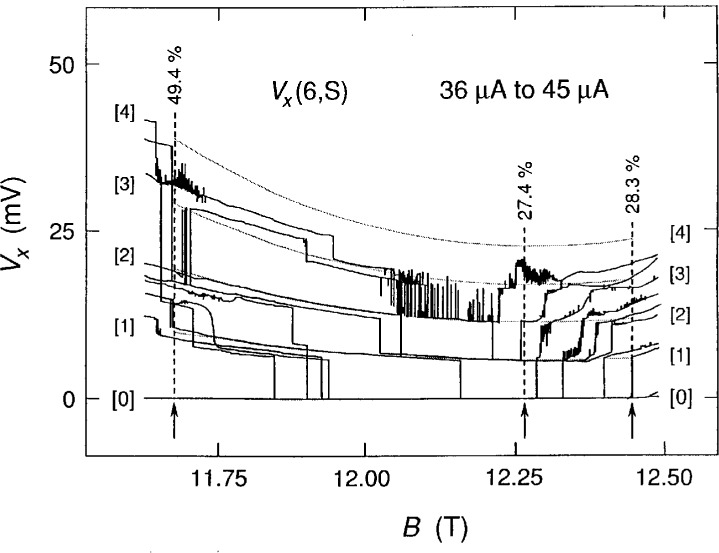
An enlarged view of part of the data shown in Fig. 1. A family of shaded curves having equal voltage spacing at each magnetic flux density value is fitted to the 41 mA data. Voltage quantization numbers are shown in brackets. The percentages of conducting electrons making transitions to higher Landau levels are indicated for three magnetic flux density values.

A portion of Fig. 1 where the *V_x_* signals are quantized is enlarged in Fig. 3, and a family of shaded curves is also displayed. These curves have equal (quantized) voltage separations at each value of magnetic flux density, but the voltage separations are allowed to vary with *B* in order to obtain smooth curves that provide the best fit to the data. The five shaded curves correspond to a *V_x_* = 0.0 mV ground state and four excited states. Quantum numbers *M* of the voltage states are labeled in brackets. The data are current-dependent for *B* less than about 12.2 T, so the shaded curves are arbitrarily fitted to the 41 μA data, which is about midway in the current range.

We use a simple black-box model [7–9] based on energy conservation arguments to interpret some aspects of the voltage quantization displayed in Fig. 3. The dissipation detected by the *V_x_*(6,S) signal is assumed to arise from transitions in which electrons occupying states of the originally full ground state Landau level are excited to states in higher Landau levels and then return to the lowest Landau level. There is an electrical energy loss per carrier for *M* Landau level transitions of *Mħω*_c_, where *ω*_c_ = eB/m* is the cyclotron angular frequency and m* is the reduced mass of the *electron* (0.068 times the free electron mass *m*_e_ in GaAs). The power loss is *I*_SD_*V_x_*, and *I*_SD_*V_x_* = *r*(2/*i*)*Mħω*_c_, where *r* is the transition rate from the ground state to the excited state and then back to the ground state, and *i* is the Hall plateau number. Thus

$$fM=\left(\frac{re}{I_{\text{SD}}}\right)M=\left(\frac{i}{2}\right)\left(\frac{m*}{\hbar }\right)\left(\frac{V_{x}}{B}\right),$$ (1)

where *f* is the ratio of the transition rate *r* within the breakdown region to the rate *I*_SD_/*e* that electrons transit the device; *f* can also be interpreted as the fraction of conducting electrons that undergo transitions.

The black-box model predicts that about 49.4 %, 27.4 %, and 28.3 % of the conducting electrons are making inter-Landau transitions for the three magnetic flux densities selected in Fig. 3, with an uncertainty of about ±1 %. The 49.4 % value is for *I*_SD_ = 41 μA, but whatever the current, large numbers of electrons seem to be making these transitions. The transition rates at 41 μA are 1.3 × 10^14^/s, 7.0 × 10^13^/s, and 7.2 × 10^13^/s, respectively for these three percentage values.

### 3.2 Maximum Electric Field

To predict the maximum value of the electric field *E*_max_ within the sample when breakdown is occurring we use the quasi-elastic inter-Landau level scattering (QUILLS) model of Heinonen, Taylor, and Girvin [12] and Eaves and Sheard [13], and the notation and coordinate system of Cage and Lavine [15]. The conducting electrons completely fill the maximum allowed number of states of the first (*N* = 0) Landau level. Wavefunctions of these states are represented in the Landau gauge as normalized products of Hermite polynomials across the sample in the *y* direction multiplied by plane waves propagating down the sample in the *x* direction. Each state undergoes cycloidal motion down the device and occupies a unique center of mass position *y*_0_ somewhere across the device width.

The confining potential and the applied current create an electric field distribution *E*(*y*) across the device [16]. If *E*(*y*) becomes sufficiently large in some portion of the device width then the Landau levels tilt enough to allow a population inversion, and electrons occupying eigenstates at positions *y*_0_ in the lowest Landau level *N* can make transitions to states of lower total energy at positions *y*_0_′ in a higher Landau level *N*′. Acoustic phonons are emitted in the *x* direction during these transitions in order to conserve energy and momentum. The electrons then emit optical phonons of total energy (*N*′ – *N*)*ħω*_c_ and return to eigenstates of the initial ground state Landau level *N*.

We can obtain a reasonable estimate of the maximum electric field by noting that the spatial extent of the *y*-axis motion of the wavefunction in Ref. [15] decays rapidly beyond the turning points of a classical harmonic oscillator whose amplitude of motion is $A_{N}=l_{B}\sqrt{2N+1}$ [13], where *l_B_* = (*ħ*/*eB*)^1/2^ is the magnetic length and the cyclotron radius of the lowest Landau level. The matrix elements of the acoustic phonon transitions become significant only when the initial and final state wavefunctions overlap [13, 17]. Transitions between the *N* and *N*′ eigenstates therefore commence when

$$(y_{0}-{y_{0}}^{\prime })\approx l_{B}\left(\sqrt{2N+1}+\sqrt{2N^{\prime }+1}\right)$$ (2)

where *N* = 0 in our case for the *i* = 2 plateau, and *N*′ *= M*. The maximum electric field is then

$$E_{\max}\approx \frac{M\hbar \omega_{c}}{e(y_{0}-{y_{0}}^{\prime })},$$ (3)

where the small contribution of the acoustic phonon transition in the numerator of Eq. (3) has been neglected.

We can use Eqs. (2) and (3) to calculate *E*_max_ at 12.25 T for the *M* = 1 transition of Fig. 3 (which is first excited at 40 μA). *E*_max_ is 1.1 × 10^6^ V/m. That happens to be the same value predicted for the *M* = 1 transition of *V_x_*(4,6) for the GaAs(8) device at 12.3 T and 215 μA [15]. Note that Eqs. (2) and (3) at first appear to be independent of current, but in reality the current must be increased to a unique value before *M* = 1 transitions are induced. Also, note that it was possible to obtain this value of *E*_max_ only because the *M* values could be uniquely identified in the breakdown data of Fig. 3.

The confining potential has large gradients near the device periphery, and the logarithmic charge redistribution potential, which arises from the applied current, also increases dramatically at one side of the device [16]. This side is determined by the magnetic field direction interacting with the conducting electrons. Therefore in our case, *E*_max_ is likely to be located somewhere along the side, between the source S and potential probe 6.

An *E*_max_ value of 1.1 × 10^6^ V/m generates a large local current density *J_x_* = *σ_xy_E*_max_ = *E*_max_/12 906.4 Ω = 85 A/m at *I*_SD_ = 40 μA. The electron drift velocity in this region of the device is then *v_x_* = *E*_max_/*B* = 8.9 × 10^4^ m/s. This electron velocity is 36 times faster than the acoustic phonon velocity *v*_s_ = 2.47 × 10^3^ m/s [18]. The value of the acoustic phonon energy *ħω*_s_ = *ħMω*_c_*v*_s_*/*(*v_x_*−*v*_s_) is 2.9 % of the total optical phonon energy *Mħω*_c_ (which is 3.4 × 10^−21^ J).

## 4. Conclusions

It is the longitudinal voltage, *V_x_*, rather than the longitudinal resistance, *R_x_*, that is quantized in breakdown of the quantum Hall effect at large applied currents. Proposed mechanisms and models for the breakdown phenomena must account for, or at least not contradict, this fact. The black-box model [6–9,15] and the quasi-elastic inter-Landau level scattering model [12,13,15,17] are consistent with this observation, and lead to predictions of the transition rates and maximum electric fields within the device.

## Footnotes

1 Certain commercial equipment, instruments, or materials are identified in this paper to foster understanding. Such identification does not imply recommendation or endorsement by the National Institute of Standards and Technology, nor does it imply that the materials or equipment identified are necessarily the best available for the purpose.
